# Xa inhibitor edoxaban ameliorates hepatic ischemia-reperfusion injury via PAR-2–ERK 1/2 pathway

**DOI:** 10.1371/journal.pone.0292628

**Published:** 2024-05-15

**Authors:** Koki Maeda, Naohisa Kuriyama, Daisuke Noguchi, Takahiro Ito, Kazuyuki Gyoten, Aoi Hayasaki, Takehiro Fujii, Yusuke Iizawa, Yasuhiro Murata, Akihiro Tanemura, Masashi Kishiwada, Shugo Mizuno

**Affiliations:** Department of Hepatobiliary Pancreatic and Transplant Surgery, Mie University Graduate School of Medicine, Tsu, Mie, Japan; Zagazig University, EGYPT

## Abstract

Hepatic ischemia-reperfusion injury causes liver damage during surgery. In hepatic ischemia-reperfusion injury, the blood coagulation cascade is activated, causing microcirculatory incompetence and cellular injury. Coagulation factor Xa (FXa)- protease-activated receptor (PAR)-2 signaling activates inflammatory reactions and the cytoprotective effect of FXa inhibitor in several organs. However, no studies have elucidated the significance of FXa inhibition on hepatic ischemia-reperfusion injury. The present study elucidated the treatment effect of an FXa inhibitor, edoxaban, on hepatic ischemia-reperfusion injury, focusing on FXa-PAR-2 signaling. A 60 min hepatic partial-warm ischemia-reperfusion injury mouse model and a hypoxia-reoxygenation model of hepatic sinusoidal endothelial cells were used. Ischemia-reperfusion injury mice and hepatic sinusoidal endothelial cells were treated and pretreated, respectively with or without edoxaban. They were incubated during hypoxia/reoxygenation *in vitro*. Cell signaling was evaluated using the PAR-2 knockdown model. In ischemia-reperfusion injury mice, edoxaban treatment significantly attenuated fibrin deposition in the sinusoids and liver histological damage and resulted in both anti-inflammatory and antiapoptotic effects. Hepatic ischemia-reperfusion injury upregulated PAR-2 generation and enhanced extracellular signal-regulated kinase 1/2 (ERK 1/2) activation; however, edoxaban treatment reduced PAR-2 generation and suppressed ERK 1/2 activation *in vivo*. In the hypoxia/reoxygenation model of sinusoidal endothelial cells, hypoxia/reoxygenation stress increased FXa generation and induced cytotoxic effects. Edoxaban protected sinusoidal endothelial cells from hypoxia/reoxygenation stress and reduced ERK 1/2 activation. PAR-2 knockdown in the sinusoidal endothelial cells ameliorated hypoxia/reoxygenation stress-induced cytotoxicity and suppressed ERK 1/2 phosphorylation. Thus, edoxaban ameliorated hepatic ischemia-reperfusion injury in mice by protecting against micro-thrombosis in sinusoids and suppressing FXa-PAR-2-induced inflammation in the sinusoidal endothelial cells.

## Introduction

Hepatic ischemia/reperfusion injury (IRI) causes liver damage during liver resection and transplantation [1]. Although many mediators have been identified as causes of hepatic IRI [2–4], its precise mechanism remains unclear. The blood coagulation cascade is activated in hepatic IRI, causing microcirculatory incompetence and cellular injury [5]. The efficacy of anticoagulant agents such as anti-thrombin Ⅲ [6] and low molecular weight heparin [7] for preventing hepatic IRI has been reported. We had previously focused on the relationship between hepatic IRI and blood coagulation cascade to report the cytoprotective effect of activated protein C in hepatic IRI [8,9].

Protease-activated receptors (PAR), comprising PAR-1 to 4, are members of a large family of seven transmembrane domain G-protein-coupled receptors, specifically activated by various proteases [10]. Recently, we revealed that thrombin-PAR-1 signaling was associated with hepatic IRI, and a PAR-1 antagonist ameliorated hepatic IRI by protecting sinusoidal endothelial cells (SECs) [11]. In addition, the significance of selective thrombin inhibitor dabigatran for sinusoidal protection against hepatic IRI was reported [12]. PAR-1 is a thrombin receptor, whereas PAR-2 is thrombin insensitive and activated by tryptase, trypsin, and the coagulation factors VⅡa and Xa [13]. PAR-2 is expressed in a wide range of human tissues and is a mediator for inflammation [14,15], cell proliferation [16], epithelial barrier function [17], and fibrosis [18]. In liver diseases, coagulation factor Xa (FXa)-PAR-2 signaling promotes hepatic inflammation [19] and liver fibrosis [20]. The liver consists of many types of cells, such as hepatocytes, SECs, Kupffer cells, hepatic stellate cells, and cholangiocytes. In the hepatic IRI, SECs are the first cells to direct the blood flow into the sinusoids and act as the primary barrier between the blood flow and hepatocytes, thus playing important protective roles in controlling vascular hemostasis and inflammation [21]. In view of this, we focused on SECs and hypothesized that FXa inhibition might protect SECs and ameliorate hepatic IRI by suppressing FXa-PAR-2 signaling-induced inflammation.

Direct oral anticoagulants (DOACs), widely used anticoagulants that directly inhibit thrombin or FXa, are superior to warfarin in terms of lower bleeding risk and lower drug-drug and drug-food interactions during liver transplantation [22]. Edoxaban is an oral, direct FXa inhibitor commonly used for the prophylaxis of venous thromboembolism and stroke prevention in patients with arterial fibrillation. The preventive effect of FXa inhibitor in various IRI models such as the kidney [23,24], heart [25], and extremities has been reported [26]. In liver diseases, FXa inhibitor reduced liver fibrosis in mice [27] and acute liver injury in a lipopolysaccharide-induced coagulopathy model in rats [28]. However, to our knowledge, no prior studies have elucidated the significance of FXa inhibition on hepatic IRI.

Therefore, this study aimed to elucidate the effect of edoxaban treatment on hepatic IRI using a partial-warm IRI model of mice and hypoxia/reoxygenation (H/R) model of hepatic SECs, with focus on the FXa-PAR-2 signaling.

## Materials and methods

### Animals

Seven- to nine-week-old male C57BL/6 mice (17–23 g; Japan SLC Inc., Hamamatsu, Japan) were used. All experiments were reviewed and approved by the Animal Care and Use Committee at Mie University Graduate School of Medicine (No. 28-9-3) and conducted in compliance with the Guidelines for Animal Experiments of Mie University Graduate School of Medicine. All animals received humane care according to the criteria outlined in the "Guide for the Care and Use of Laboratory Animals" prepared by the National Academy of Sciences and published by the National Institute of Health.

### Partial-warm hepatic IRI model

A hepatic partial-warm IRI model was established in mice [21]. Mice were anesthetized with isoflurane, and their livers were exposed using midline laparotomy. The arterial and portal venous blood supplies were interrupted to the cephalad lobes of the liver for 60 minutes using an atraumatic clip. The right hepatic and caudate lobes were perfused to prevent intestinal congestion. After 60 minutes of ischemia, the clip was removed, initiating hepatic reperfusion. At 4 hours after reperfusion, the mice were euthanized by overdose administration of isoflurane to collect blood and liver tissues. In order to alleviate the suffering of mice, all invasive procedures were performed under appropriate anesthesia.

### Dose determination of edoxaban for hepatic IRI study of mice *in vivo*

A potent and highly selective FXa inhibitor, edoxaban tosylate monohydrate (Ex) (MedChem Express, Monmouth Junction, NJ), an orally active prodrug of edoxaban, was administered to mice through oral gavage. Ex was dissolved in 0.5 w/v% methylcelluloses 400 solution (0.5% MC; FUJIFILM Wako Pure Chemical Industries, Ltd., Osaka, Japan) [29]. In our model, Ex was administered orally to mice because it must undergo chemical conversion by metabolic processes through oral intake for activation. Previously, sufficient anticoagulant effects on mice were observed at 60 minutes after oral administration of 10 mg/kg Ex [30], and renal protective effects for diabetic nephropathy mice model were observed with 50 mg/kg/day [31,32]. Following these reports, to examine the effect of Ex treatment in our mice hepatic IRI model, based on serum alanine transaminase (ALT) levels at 4 hours after reperfusion, we set the doses of Ex as 3, 10, 30, and 50 mg/kg, and administered them orally 60 minutes before ischemia (n = 5 per group). A dose of 50 mg/kg had a significant effect: 1,008 [800–1,474] IU/L in controls (Vehicle) vs. 89 [35–242] IU/L in the edoxaban group (*p* = 0.05) (S1 Fig). Accordingly, we decided to administer 50 mg/kg of Ex.

### Experimental groups of mice

All mice were randomly allocated to two IRI groups (n = 6 per group). One hour before ischemia, the edoxaban and vehicle groups received oral administration of 50 mg/kg of Ex and vehicle (0.5% MC equivalent to that used to dissolve Ex), respectively. These mice underwent the surgery described above.

### Measurement of serum transaminases

Serum aspartate transaminase (AST) and ALT levels were measured using a commercially available kit (Test Wako for transaminase; Wako Pure Chemical Industries Ltd., Osaka, Japan) following the manufacturer’s instructions.

### Histology

Liver specimens were fixed in a 10% buffered formalin solution, embedded in paraffin, and processed for hematoxylin and eosin staining. The histological severity of hepatic IRI was graded using a modified Suzuki’s score [33]. The results were evaluated by averaging 10 scores in 20 high-power fields per section in a blinded manner.

### Immunohistochemistry

Liver specimens embedded in Tissue-Tek OCT compound (Miles, Elkhart, IN) and snap-frozen in liquid nitrogen were used for immunostaining [34]. Primary antibodies against Ly6G (BioLegend, San Diego, CA) and mouse macrophage antigen-1 (MAC-1; BioLegend, San Diego, CA) were used at a dilution of 1:500. The results were evaluated by averaging 10 counts of the number of Ly6G and MAC-1 positive cells in 20 high-power fields per section in a blinded manner.

### Immunofluorescence analysis

Liver specimens embedded in paraffin were deparaffinized and rehydrated. The monoclonal antibody against mouse fibrin (clone 59D8, MABS2155; EMD Millipore, Bedford, MA) and polyclonal antibody against rabbit cluster of differentiation 31 (CD31; Santa Cruz Biotechnology, CA) at dilutions of 1:300 and 1:50, respectively, were used as primary antibodies. Fluorescent signals were detected by Alexa Fluor 488 (green)-labeled and Alexa Fluor 594 (red)-labeled secondary antibodies. VECTASHIELD mounting media with DAPI (Vector Laboratories; Burlingame, CA) were used for nuclear staining. Slides were observed using a fluorescence microscope (BX51; Olympus, Tokyo, Japan).

### Terminal transferase dUTP nick end labeling (TUNEL) staining

TUNEL staining was performed to evaluate apoptotic cells of mice (n = 6 in each group) *in vivo*, using the In Situ Cell Death Detection Kit (Cat No. 11684795910; Roche Diagnostics, Temecula, CA), following the manufacturer’s instructions. Paraffin-embedded liver tissue sections were deparaffinized and rehydrated, followed by 350 W microwave irradiation before the TUNEL reaction. All samples were analyzed using a fluorescence microscope. The results were evaluated by averaging 10 counts of TUNEL-positive cells in 20 high-power fields per section in a blinded manner.

### Western blot analysis

Total protein from the whole liver *in vivo* or cell lysate *in vitro* were extracted, and western blot analyses were performed with equal protein loading [21]. In brief, protein-transferred polyvinylidene fluoride membranes (EMD Millipore, Bedford, MA) were incubated overnight with specific primary antibodies against fibrin (clone 59D8, MABS2155; EMD Millipore, Bedford, MA), cleaved-caspase 3 (#9664; Cell Signaling Technology, Beverly, MA), pro-caspase 3 (#14220; Cell Signaling Technology), phospho-extracellular signal-regulated kinase (ERK) 1/2 (#9101; Cell Signaling Technology), total-ERK 1/2 (#9102; Cell Signaling Technology), PAR-2 (sc-13504; Santa Cruz Biotechnology, CA), and β-actin (#4967; Cell Signaling Technology) at 4°C, followed by a horseradish peroxidase-linked secondary antibody for 2 hours, at 22–26°C. After development, the membranes were stripped and reblotted with β-actin antibody. The immunoreactive bands were detected using LuminoGraph (Atto, Tokyo, Japan). The intensities were quantified using a densitometry tool (CS Analyzer 4 version 2.2.1; Atto, Tokyo, Japan) and normalized to the internal control (β-actin protein).

### RNA extraction and real-time quantitative polymerase chain reaction (PCR)

We evaluated mRNA levels of tumor necrosis factor (TNF)-α, interleukin (IL)-6, B-cell lymphoma (bcl)-2, monocyte chemotactic protein (MCP)-1, CXC motif chemokine ligand (CXCL)-2, CXCL-10, PAR-2, and β-actin using TaqMan® Gene Expression Assays (Applied Biosystems, Foster City, CA), following the manufacturer’s instructions. The cDNA prepared from total RNA extracted from the livers was subjected to real-time quantitative PCR on a StepOne Real-Time PCR System (Applied Biosystems). The primer/probe pairs used in this study were from Applied Biosystems: TNF-α: Mm00443260_g1, IL-6: Mm00446190_m1, bcl-2: Mm00477631_m1, PAR-2: Mm00433160_m1, MCP-1: Mm99999056_m1, CXCL-2: Mm00436450_m1, CXCL-10: Mm00445235_m1, and β-actin: Mm00607939_s1. β-actin was used as a normalization control.

### Cell culture of SECs *in vitro* hypoxia-reoxygenation models

Human hepatic SECs were purchased from ScienCell Research Laboratories, San Diego, CA. The hypoxia-reoxygenation (H/R) models were established using an AnaeroPack jar system (Mitsubishi Gas Chemical Co., Tokyo, Japan) [11]. In brief, all cells (0.5 × 10^4^/well) were seeded and cultured in endothelial cell medium (ScienCell Research Laboratories, San Diego, CA) for SECs on a 24-well collagen-coated plate at 37°C with 5% CO_2_ for 48 hours. After semi-confluence, these cells cultured in the serum-starved medium were exposed to hypoxic conditions (< 0.1% O_2_) for 90 minutes, followed by reoxygenation for 4 hours.

### *In vitro* study of experimental SECs groups to elucidate the effects of edoxaban for H/R

Edoxaban (MedChem Express, Monmouth Junction, NJ) was used for the *in vitro* study. SECs were allocated to two H/R groups and then cultured (n = 5 per group). The edoxaban and vehicle groups were pretreated with 1 μM of edoxaban in endothelial cell medium and vehicle (0.1% DMSO equivalent to that used to dissolve edoxaban) for 1 hour. Following the pretreatment, the SECs in both groups were exposed to 90-minute hypoxia and 4 hours reoxygenation using an AnaeroPack jar system. A dose of 1 μM had a significant effect (*p* = 0.008), we decided to use 1 μM of edoxaban (S2 Fig).

### *In vitro* studies of experimental SECs groups to elucidate the influence of coagulation factor Xa and ERK 1/2 signaling pathway

SECs were allocated to two groups (n = 5 in each group). The FXa group was incubated with 0.5 IU/L of human FXa (#HCXA-0060; Haematologic Technologies, Essex Junction, VT) in endothelial basal medium for 4 hours, while the control group was incubated with endothelial basal medium alone for the same time. The dose and incubation time of FXa were determined according to a previous report [35].

ERK1/2 signaling pathway was inhibited using a specific MAPK inhibitor (PD98059; Sigma Aldrich, St.Louis, MO). The PD98059 and vehicle groups were pretreated with 10 μM of PD98059 in endothelial cell medium and vehicle (0.1% DMSO equivalent to that used to dissolve PD98059) for 1 hour, followed by H/R as mentioned above. The dose and incubation time were determined according to a previous report [11].

### Small interfering RNA (siRNAs) preparation and transfection

Pre-designed and validated siRNAs directed against PAR-2 and negative control were used (Life Technologies, Carlsbad, CA). The siRNA sequences targeting PAR-2 were 5’-CAAUAGAUCCUCUAAAGGAtt-3’ (sense) and 5’-UCCUUUAGAGGAUCUAUUGgt-3’ (antisense). Silencer^®^ Negative Control #1 siRNA was used for negative controls. SECs were transfected for 48 hours with 5 nM of siRNA using lipofectamine RNAiMAX reagent according to the manufacturer’s instructions (Life Technologies, Carlsbad, CA) prior to H/R stress. Negative control was transfected in parallel (n = 5 per group). PAR-2 expression levels in siRNA transfectants were measured by western blotting.

### Lactate dehydrogenase assay

Cell cytotoxicity was assessed by measuring lactate dehydrogenase (LDH) levels in the supernatant using a Cytotoxicity LDH Assay Kit-WST (Dojindo, Japan), following the manufacturer’s instructions.

### Data analysis

Data were expressed as medians and interquartile ranges. Data distribution was analyzed using the Shapiro-Wilk test. Differences between groups were analyzed using the Mann-Whitney U test in SPSS (version 26; IBM Corp, Armonk, NY, USA). Multiple comparisons were performed using one-way analysis of variance with the Welch test followed by the Games-Howell test for parametric data, and the Kruskal-Wallis test followed by the Steel-Dwass test for non-parametric data using R (version 3.6.1; R Foundation for Statistical Computing, Vienna, Austria); *p* < 0.05 was considered statistically significant.

## Results

### Edoxaban mitigates hepatocellular injury after ischemia-reperfusion, *in vivo*

**Fig 1:**
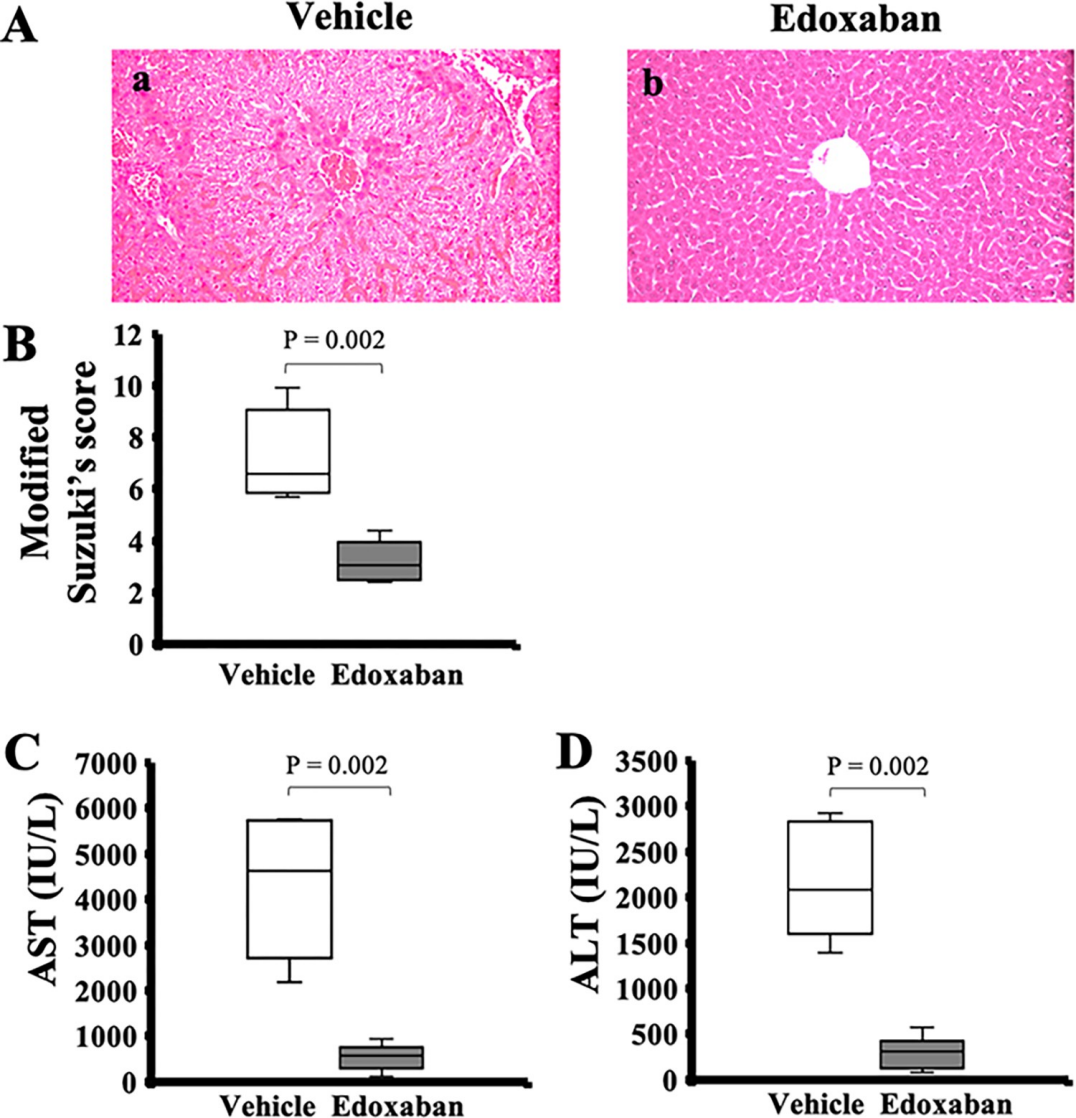
Liver histology and transaminases after hepatic ischemia-reperfusion injury (IRI) with or without edoxaban. (A) IRI livers treated with (a) vehicle showed sinusoidal vascular congestion and diffuse cell degeneration from the periportal to the pericentral area. In contrast, sinusoidal vascular congestion and diffuse cell degeneration were suppressed in the (b) edoxaban group (original magnification × 200). (B) The modified Suzuki’s score in the edoxaban group was significantly lower than that in the vehicle group (n = 6 per group). Serum (C) AST and (D) ALT levels were significantly lower in the edoxaban group than the vehicle group. (n = 6 per group). P-value from the Mann-Whitney U test.

In histology, sinusoidal vascular congestion and diffuse cell degeneration from the periportal to the pericentral area were observed in the vehicle group (Fig 1A-a). Meanwhile, sinusoidal vascular congestion and diffuse cell degeneration were suppressed in the edoxaban group (Fig 1A-b). The modified Suzuki’s score was significantly lower in the edoxaban group than the vehicle group (6.6 [5.9–8.4] in the vehicle, 3.1 [2.6–3.7] in the edoxaban, *p* = 0.002; Fig 1B).

Serum AST and ALT levels were significantly lower in the edoxaban group than the vehicle group (AST: 4613 [3303–5452] IU/L in the vehicle, 572 [379–701] IU/L in the edoxaban, *p* = 0.002; ALT: 2087 [1676–2718] IU/L in the vehicle; 313 [170–375] IU/L in the edoxaban, *p* = 0.002; Fig 1C and 1D).

### Edoxaban treatment reduced fibrin deposition in hepatic sinusoids

**Fig 2:**
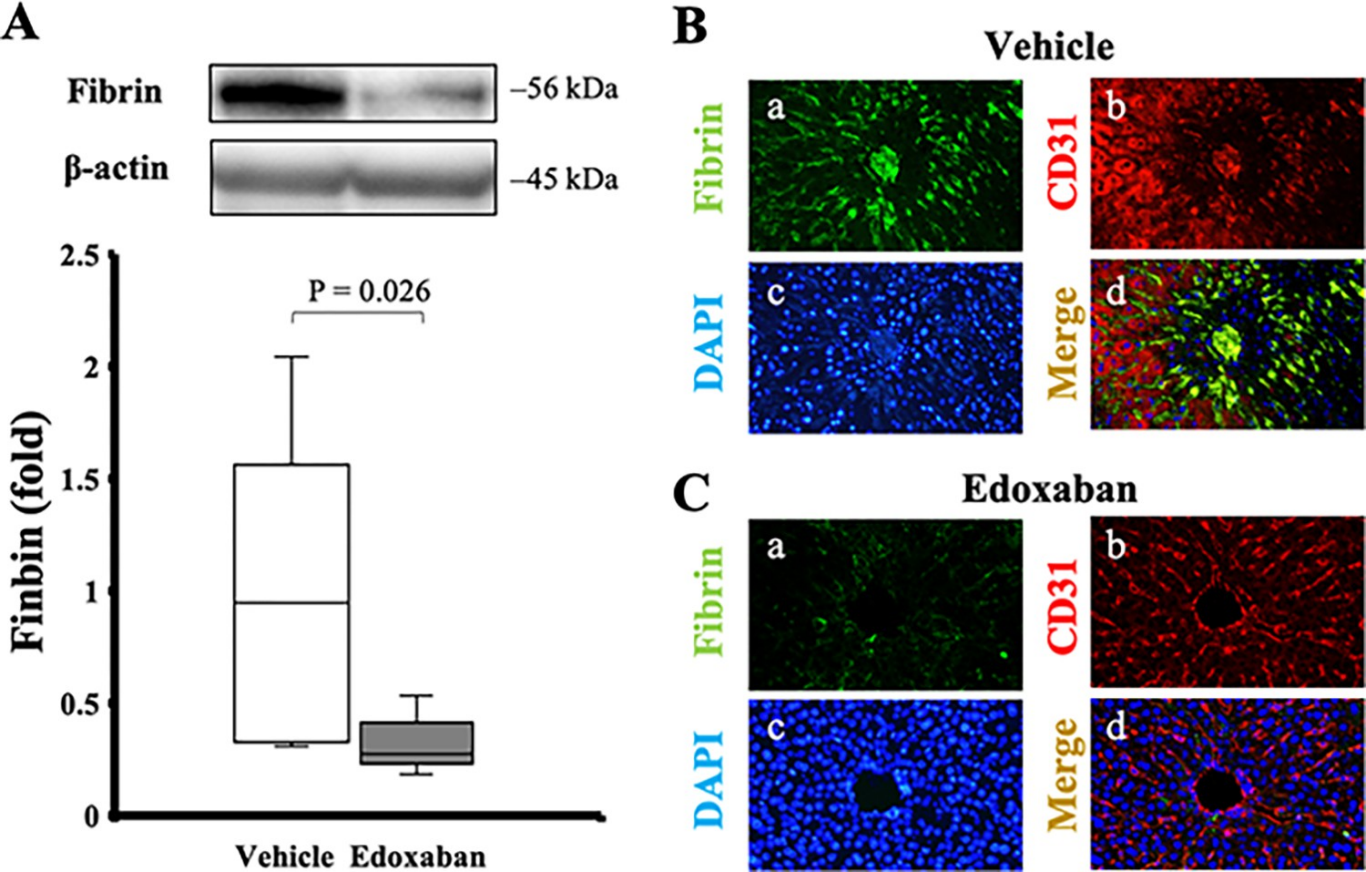
Fibrin deposition in the liver. (A) Edoxaban treatment significantly reduced fibrin generation in the liver tissue (n = 6 per group). Immunofluorescent analysis in IRI livers treated with (B) vehicle and (C) edoxaban (original magnification × 200): a, DAPI in blue; b, CD31 in red; c, fibrin in green; d, merged image. (C-d) Fibrin deposition on hepatic sinusoids was ameliorated by edoxaban. P-value from the Mann-Whitney U test.

In western blot analysis, the generation of fibrin was significantly suppressed in the edoxaban group compared with the vehicle group (0.95 [0.43–1.36] in the vehicle, 0.28 [0.25–0.36] in the edoxaban, *p* = 0.026; Fig 2A). Immunofluorescent analysis revealed that the deposition of fibrin in sinusoids was decreased in the edoxaban group compared with the vehicle group (Fig 2B and 2C).

### Edoxaban ameliorates inflammatory and apoptotic reactions induced by hepatic ischemia-reperfusion injury

**Fig 3:**
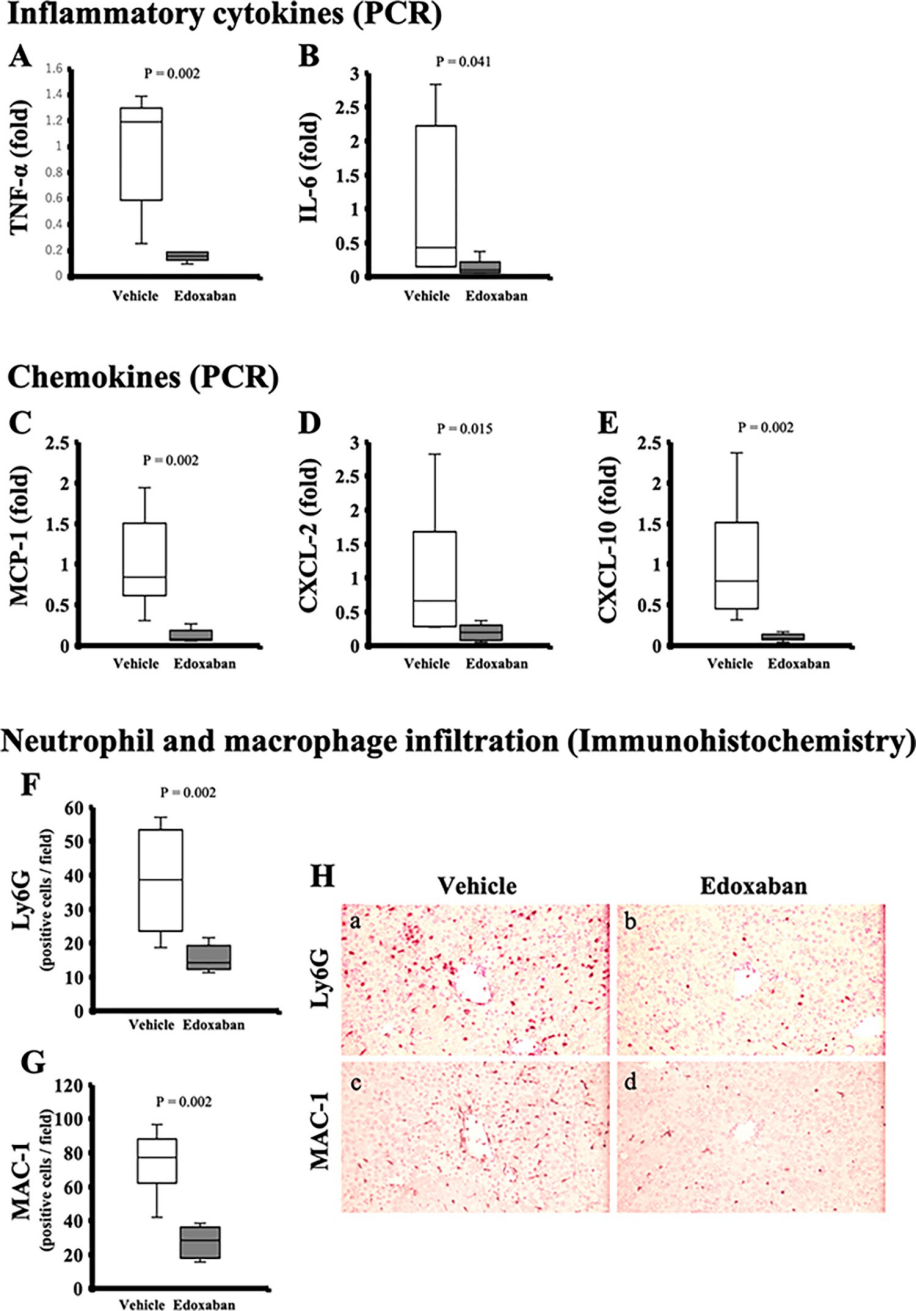
Anti-inflammatory effects of edoxaban treatment in hepatic ischemia-reperfusion injury. Real-time PCR analysis showed that the expression of inflammatory cytokines, such as (A) TNF-α and (B) IL-6, and chemokines, such as (C) MCP-1, (D) CXCL-2 and (E) CXCL-10 were significantly lower in the edoxaban group (n = 6 per group). The average number of (F) Ly6G and (G) MAC-1 positive cells were significantly lower in the edoxaban group (n = 6 in each group). (H) Immunohistochemistry of Ly6G and MAC-1 in the vehicle group and the edoxaban group (original magnification × 200). P-value from the Mann-Whitney U test.

The expression of inflammatory cytokines, such as TNF-α and IL-6, and chemokines, such as MCP-1, CXCL-2, and CXCL-10 were significantly lower in the edoxaban group (TNF-α/β-actin: 1.2 [0.8–1.3] in the vehicle, 0.2 [0.1–0.2] in the edoxaban, *p* = 0.002; IL-6/β-actin: 0.43 [0.18–1.66] in the vehicle, 0.09 [0.07–0.15] in the edoxaban, *p* = 0.041; MCP-1/β-actin: 0.8 [0.7–1.2] in the vehicle, 0.1 [0.1–0.1] in the edoxaban, *p* = 0.002; CXCL-2/β-actin: 0.7 [0.4–1.1] in the vehicle, 0.2 [0.1–0.3] in the edoxaban, *p* = 0.015; CXCL-10/β-actin: 0.8 [0.6–1.1] in the vehicle, 0.1 [0.1–0.1] in the edoxaban, *p* = 0.002; Fig 3A–3E). Immunohistochemistry of Ly6G and MAC-1 positive cells showed diffused neutrophil (Ly6G positive) and macrophage (MAC-a positive) infiltration in the vehicle group; on the other hand, the number of infiltrated those inflammatory cells was decreased in the edoxaban group (Ly6G: 38.7 [27.4–49.9] in the vehicle, 14.3 [12.8–17.6] in the edoxaban, *p* = 0.002; MAC-1: 77.4 [69.9–84.5] in the vehicle, 28.4 [20.9–33.9] in the edoxaban, *p* = 0.002; Fig 3F–3H).

**Fig 4:**
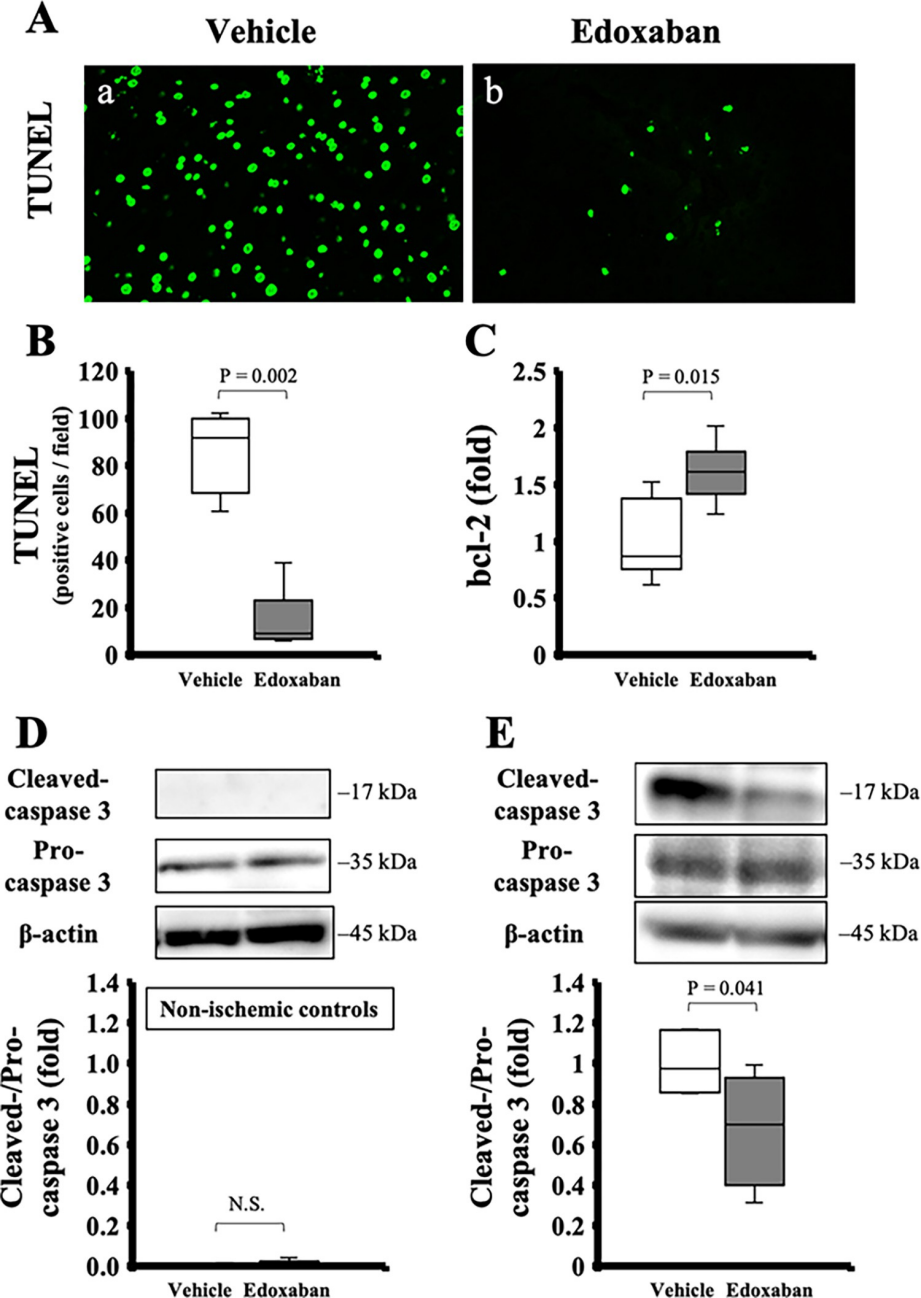
Antiapoptotic effect of edoxaban treatment for hepatic ischemia-reperfusion injury. (A) TUNEL staining in (a) the vehicle group and (b) the edoxaban group (original magnification × 200). (B) The average number of TUNEL-positive cells was significantly lower in the edoxaban group (n = 6 per group). (C) Real-time PCR analysis revealed that the generation of bcl-2 was significantly higher in the edoxaban group (n = 6 per group). (D, E) According to western blot analysis, activation of caspase 3 was significantly lower in the edoxaban group (n = 6 per group). P-value from the Mann-Whitney U test.

The number of TUNEL-positive cells was significantly lower in the edoxaban group (91.6 [75.3–98.2] in the vehicle, 9.0 [7.5–15.6] in the edoxaban, *p* = 0.002; Fig 4A and 4B). Additionally, the generation of bcl-2 was significantly higher (bcl-2/β-actin: 0.9 [0.8–1.2] in the vehicle, 1.6 [1.5–1.7] in the edoxaban, p = 0.015; Fig 4C), and activation of caspase 3 was significantly lower in the edoxaban group (cleaved-caspase 3/pro-caspase 3: 1.0 [0.9–1.1] in the vehicle, 0.7 [0.5–0.9] in the edoxaban, p = 0.041; Fig 4E).

### Hepatic IRI upregulated PAR-2 expression and generation

In the *in vivo* hepatic IRI model, PAR-2 gene expression was significantly increased after IRI by real-time PCR, than in the control group (PAR-2/β-actin: 1.6 [1.5–2.3] after IRI, 1.0 [0.9–1.0] in the control, *p* = 0.008; S3A Fig), and its generation was also significantly increased in IRI by western blot analysis (PAR-2/β-actin: 2.2 [1.6–2.4] after IRI, 0.8 [0.7–1.2] in the control, *p* = 0.032; S3B Fig).

In the H/R model of SECs *in vitro*, PAR-2 generation was also significantly increased after H/R by western blot analysis (PAR-2/β-actin: 3.1 [1.7–3.1] in after H/R, 1.2 [1.0–1.2] in the control, *p* = 0.008; S3C Fig).

### Edoxaban treatment reduced PAR-2 and ERK 1/2 generation *in vivo*

**Fig 5:**
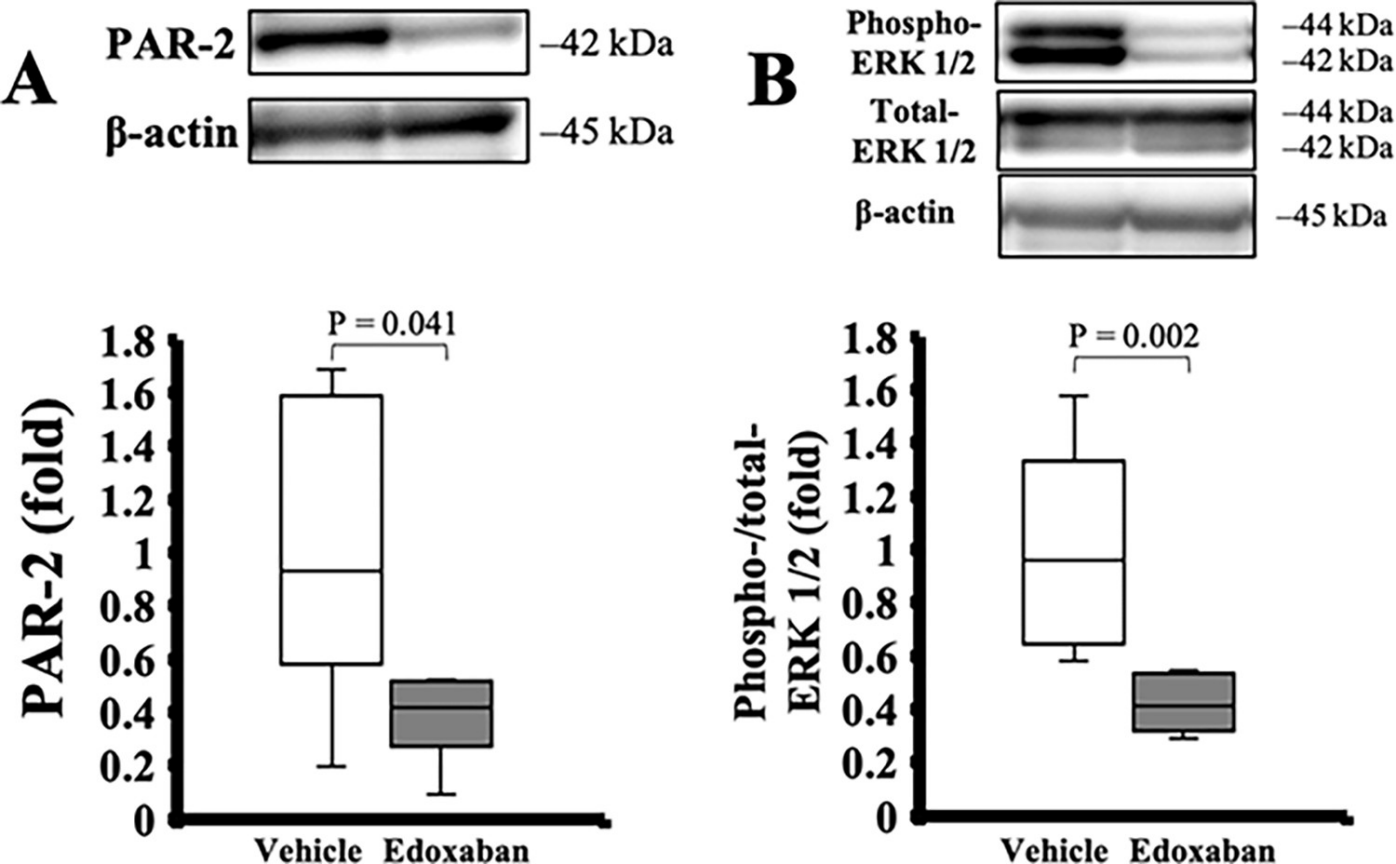
Influence of edoxaban treatment on cell signaling after hepatic ischemia-reperfusion injury *in vivo*. Edoxaban treatment significantly reduced (A) PAR-2 generation and (B) phosphorylation of ERK 1/2 in the liver after IRI (n = 6 per group). P-value from the Mann-Whitney U test.

Western blot analysis revealed that edoxaban treatment significantly reduced PAR-2 and ERK 1/2 phosphorylation in liver tissue, compared with vehicle *in vivo* (PAR-2/β-actin: 0.9 [0.7–1.4] in the vehicle, 0.4 [0.3–0.5] in the edoxaban, p = 0.041, phospho-/total-ERK 1/2: 1.0 [0.7–1.2] in the vehicle, 0.4 [0.3–0.5] in the edoxaban, p = 0.002, Fig 5A and 5B).

### Edoxaban protected SECs from H/R and FXa-induced injury and reduced ERK 1/2 phosphorylation *in vitro*

**Fig 6:**
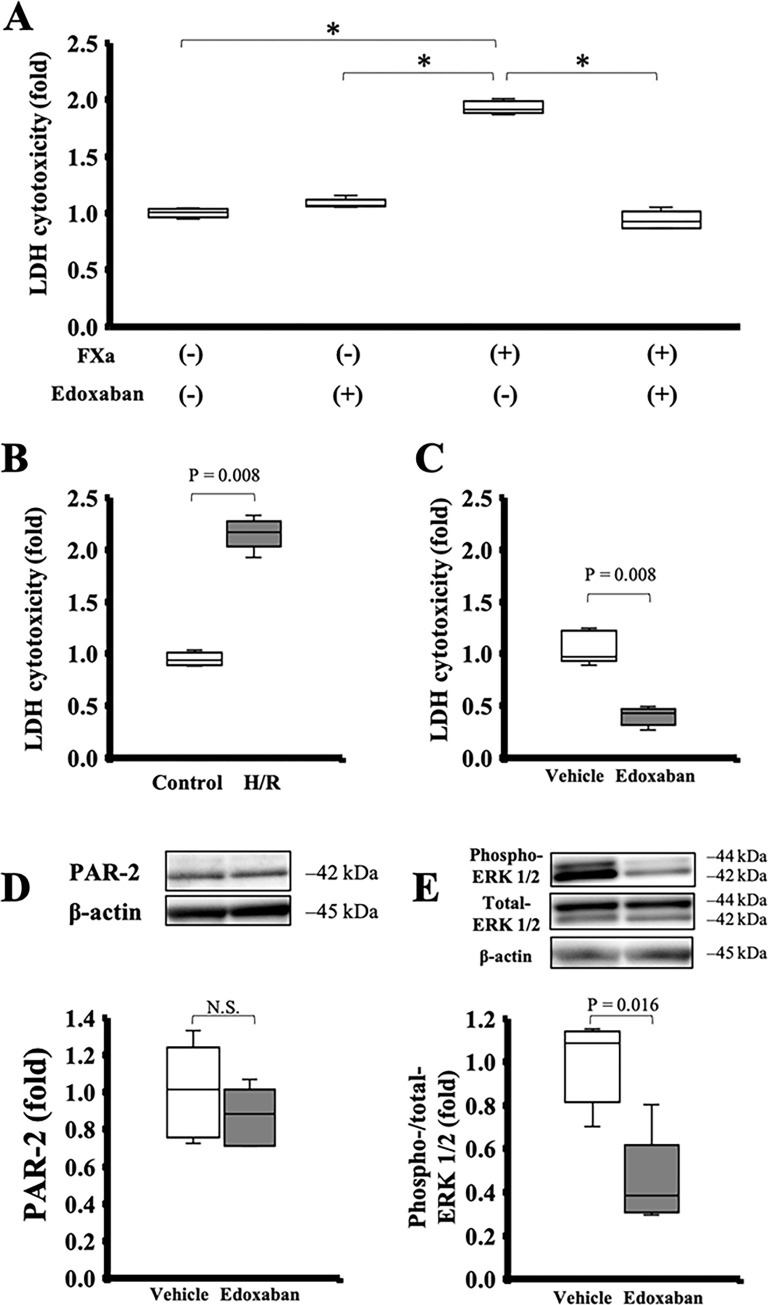
The direct effect of edoxaban treatment on hepatic sinusoidal endothelial cells (SECs) *in vitro* hypoxia/reoxygenation (H/R) models. (A) FXa increased LDH cytotoxicity levels in the hepatic SECs; however, edoxaban treatment significantly ameliorated FXa-induced cytotoxicity (n = 5 per group). P-value from the Kruskal-Wallis test followed by the Steel-Dwass test. *p < 0.05. In H/R models of hepatic SECs (n = 5 per group), (B) LDH cytotoxicity was significantly increased after H/R: however, (C) edoxaban treatment significantly decreased LDH cytotoxicity levels and (E) reduced phosphorylation of ERK 1/2 (n = 5 per group). (D) Edoxaban treatment did not affect the PAR-2 generation in the hepatic SECs after H/R (n = 5 per group). P-value from the Mann-Whitney U test (B–E).

LDH cytotoxicity levels in the supernatant of SECs incubated with FXa were significantly higher than in the controls; however, edoxaban significantly ameliorated the cytotoxicity induced by FXa (Fig 6A). LDH cytotoxicity was also significantly increased after H/R (2.2 [2.1–2.2] in after H/R, 0.9 [0.9–1.0] in the control, *p* = 0.008; Fig 6B); however, edoxaban also significantly ameliorated cytotoxicity induced by H/R stress (1.0 [1.0–1.2] in the vehicle, 0.4 [0.4–0.4] in the edoxaban, *p* = 0.008, Fig 6C) and reduced ERK 1/2 phosphorylation, compared with vehicle (phospho-/total-ERK 1/2: 1.1 [0.9–1.1] in the vehicle, 0.4 [0.3–0.4] in the edoxaban, *p* = 0.016, Fig 6E). The generation of PAR-2 was not reduced by edoxaban in H/R model of SECs (PAR-2/β-actin: 1.0 [0.8–1.1] in the vehicle, 0.9 [0.7–1.0] in the edoxaban, *p* = 0.31, Fig 6D).

### PAR-2 knockdown protected SECs from H/R injury by inhibiting ERK 1/2 pathway

**Fig 7:**
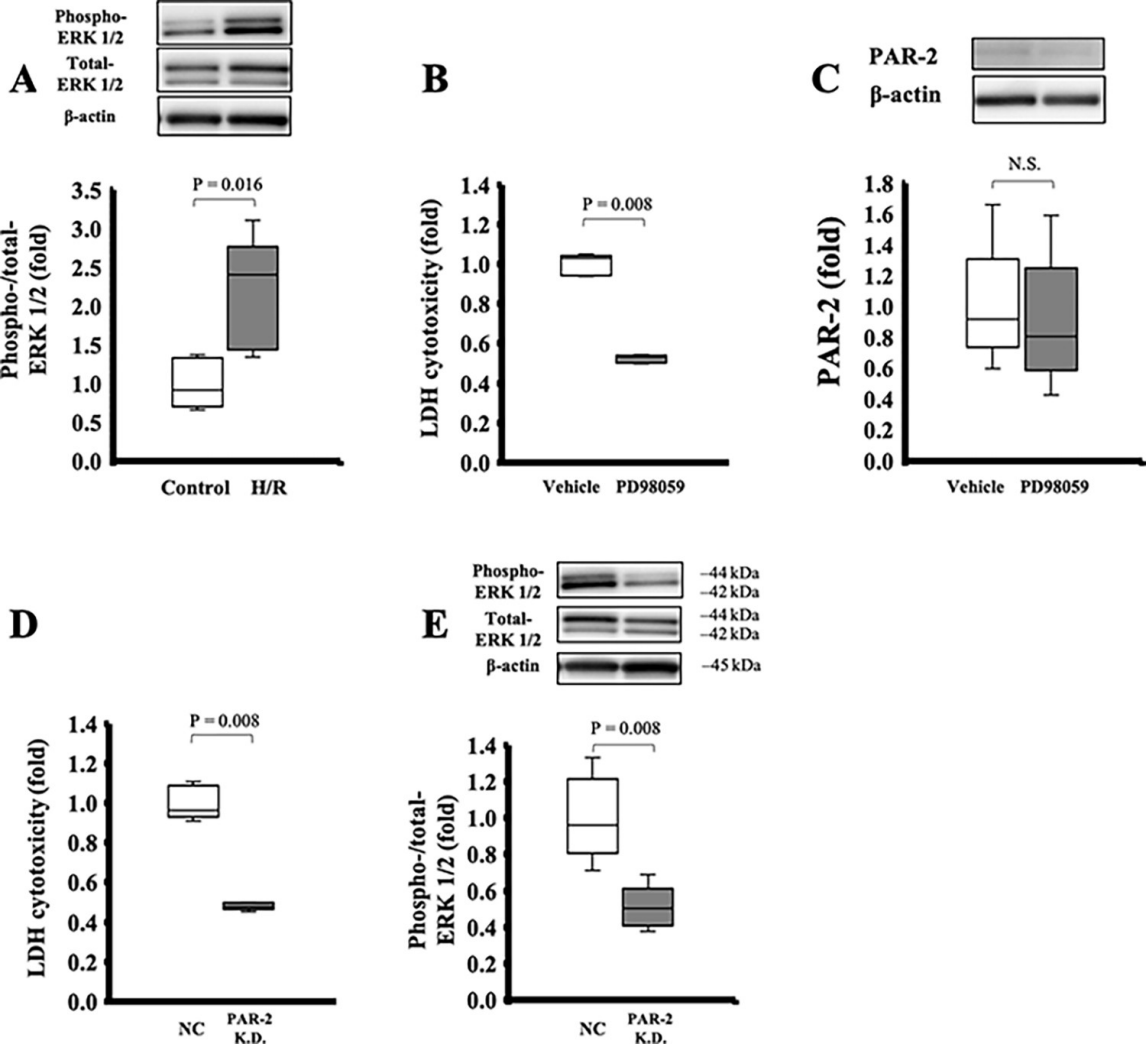
PAR-2 knockdown ameliorated cytotoxicity induced by hypoxia/reoxygenation (H/R) stress and suppressed ERK1/2 signaling. (A) H/R stress significantly increased ERK 1/2 phosphorylation in SECs. (B, C) PD98059 significantly ameliorated cytotoxicity induced by H/R stress without downregulating PAR-2. (D) PAR-2 knockdown significantly ameliorated the cytotoxicity induced by H/R stress. (E) In western blot analysis, ERK1/2 phosphorylation was significantly suppressed in the PAR-2 knockdown model. P-value from the Mann-Whitney U test.

H/R stress significantly increased ERK 1/2 phosphorylation in SECs (0.9 [0.8–1.3] in the control, 2.4 [1.5–2.4] in H/R, *p* = 0.016, Fig 7A). PD98059 significantly ameliorated cytotoxicity induced by H/R stress (1.0 [0.9–1.0] in the vehicle, 0.5 [0.5–0.5] in the PD98059, *p* = 0.008, Fig 7B) without downregulating PAR-2 (0.9 [0.9–1.0] in the vehicle, 0.9 [0.8–1.6] in the PD98059, *p* = 0.421, Fig 7C).

PAR-2 knockdown significantly ameliorated the cytotoxicity induced by H/R stress (1.0 [1.0–1.1] in the negative control, 0.5 [0.5–0.5] in PAR-2 knockdown, *p* = 0.008, Fig 7D). ERK 1/2 generation was significantly suppressed in the PAR-2 knockdown model (1.0 [0.9–1.1] in the negative control, 0.5 [0.4–0.5] in PAR-2 knockdown, p = 0.008; Fig 7E).

## Discussion

The present study revealed for the first time that a direct FXa inhibitor, edoxaban, significantly ameliorated hepatic IRI by inhibiting micro-thrombosis in sinusoids and FXa-PAR-2-induced inflammatory reaction in SECs. Edoxaban significantly attenuated fibrin deposition in the sinusoids, decreased serum transaminase levels, improved histological damage, suppressed the expression of inflammatory cytokines and inflammatory cell infiltration, and attenuated apoptotic reaction, *in vivo*. Edoxaban also suppressed PAR-2 generation and ERK 1/2 activation, activated after IRI. In addition, in our *in vitro* study using H/R model of SECs, both edoxaban and PAR-2 knockdown attenuated cytotoxicity and ERK 1/2 activation induced by H/R stress.

We previously reported the significance of dabigatran, a highly selective thrombin inhibitor, for sinusoidal protection against hepatic IRI [12]. Therefore, in the present study, we focused on FXa, a protease-activated upstream of thrombin in the coagulation cascade and a selective inhibitor of edoxaban. As per our previous report, edoxaban also attenuated fibrin deposition in the sinusoids in the hepatic IRI model of mice. This result suggested that the anticoagulant effect of edoxaban contributed to the maintenance of microcirculation in the liver from IRI, resulting in attenuating hepatic IRI injury.

The anti-inflammatory effect of edoxaban also contributes to protection against hepatic IRI. In the rat hepatic IRI model, neutrophil elastase and oxygen radicals enhanced MCP-1 and cytokine-induced neutrophil chemoattractant (CINC) expression; and DX-9065a, a prototype of edoxaban, which could only be administered parenterally, reduced MCP-1 and CINC expression [36–38]. In the present study, we revealed for the first time that edoxaban had an anti-inflammatory effect by suppressing the expression of inflammatory cytokines and chemokines and the inflammatory cell infiltration in the liver after IRI. In addition, we also showed that edoxaban had an antiapoptotic effect through the upregulation of bcl-2 expression and the reduction of caspase-3 activations.

PAR-2 is located on various organs such as the stomach, small and large intestines, pancreas, liver, kidney, and eyes, and has various pathophysiological roles [39,40]. PAR-2 is considered to be expressed in the hepatocytes, SECs, Kupffer cells, cholangiocytes, and hepatic stellate cells in the liver [20]. On the other hand, Piran *et al*. (2016) [41], reported that PAR-2 was not detectable in the normal liver but markedly upregulated after liver injury by CCl_4_. In the present study, PAR-2 was expressed in both the normal liver and SECs, and they were significantly upregulated after IRI and H/R stress.

The role of PAR-2 in the cells that consist of the liver has been shown in various experimental models. Kupffer cells have been reported to have an important role in the acute inflammatory response via FXa-PAR-2 pathway, and several studies have revealed that the hepatic stellate cells were activated to induce fibrosis by FXa-PAR-2 pathway and deactivated by FXa-inhibitor [20,42]. Although the functions of PAR-2 on the hepatocytes and SECs were unclear, several *in vitro* studies have revealed that FXa activates PAR-2 on the vascular endothelial cells and promotes multiple pro-inflammatory reactions [43,44]. In addition, several *in vivo* IRI models have shown that PAR-2 signaling is related to pro-inflammatory reactions in the intestinal [15], renal [23], hepatic [45], and cardiac IRI [46]. Furthermore, the efficacy of FXa inhibitors for attenuating FXa-PAR-2 signaling-induced inflammation *in vitro* [47] and *in vivo* [24,27,31] has been reported. In the present study, FXa, which increased after H/R stress in the SECs, significantly increased LDH cytotoxicity; however, this toxic effect was attenuated in both the edoxaban pretreated H/R model and PAR-2 knockdown model of SECs. These results suggested that FXa-PAR-2 signaling activated pro-inflammatory reactions, and FXa inhibition reduced the pro-inflammatory reactions by suppressing FXa-PAR-2 signaling in the SECs.

PAR-2 induces multiple signaling pathways after coupling with heterodimeric G proteins. PAR-2 activation induces an increase in intracellular calcium along with the production of inositol triphosphate (IP3) and diacylglycerol, and it also activates the nuclear factor—κB (NF-κB) pathway and mitogen-activated protein kinase cascade, especially in ERK 1/2 [13,48]. The FXa-PAR-2-induced inflammatory reactions were activated through ERK 1/2 in the cardiomyocytes [49] and alveolar epithelial cells [47]. In the liver, Borensztajn *et al*. (2008) [50] reported that FXa-PAR-2 signaling enhanced ERK 1/2 activation and provoked inflammation and fibrosis. Therefore, we focused on ERK 1/2 and revealed that its activation was induced in the liver after hepatic IRI *in vivo* and in the SECs after H/R stress. In addition, H/R stress-induced cytotoxicity was attenuated in the PD98059 pre-treated model, and cytotoxicity and ERK 1/2 activation were attenuated in the PAR-2 knockdown model of SECs. These results suggested that FXa-PAR-2 signaling induced ERK 1/2 activation and enhanced pro-inflammatory reactions in the SECs after H/R stress.

In the present study, edoxaban downregulated PAR-2 expression, suppressed ERK 1/2 phosphorylation, and reduced inflammatory reactions resulting in attenuating hepatic IRI *in vivo*. This suggests that edoxaban suppressed inflammation after hepatic IRI through the inhibition of FXa-PAR-2 signaling in the SECs, considering the result of the present *in vitro* studies.

In the potential clinical settings of hepatic surgery or liver transplantation, edoxaban should be administered prior to reperfusion to protect SECs against reperfusion stress and prevent activation of the coagulation cascade. In hepatic IRI, most SECs remain alive during the period of ischemia but rapidly die on reperfusion, resulting in disruption of the endothelial wall and leading to activation of the coagulation cascade [51]. Once the coagulation cascade has been activated, the effect of edoxaban should be limited. However, the systemic administration of edoxaban has the potential risk for intraoperative bleeding; therefore, further considerations are required to apply the clinical settings for hepatic surgery. As for liver transplantation, administration of FXa-inhibitor to the donor’s liver with cold storage solutions might be effective in ameliorating hepatic IRI. In addition, FXa-inhibitor might have the potential to be used as an anticoagulative agent for reducing hepatic IRI during normothermic machine perfusion for the donor’s liver instead of heparin.

There are several limitations to the present study. First, PAR-2 knockout mice are necessary for confirming whether FXa-PAR-2 signaling actually induced an inflammatory response in the hepatic IRI model *in vivo*. Second, the dose of edoxaban in the present study was much higher than the clinical dose. Third, although the dabigatran treatment did not attenuate cytotoxicity by H/R stress in our previous hepatocyte model [12], hepatocytes should be evaluated along with hepatic SECs to test whether the cytoprotective effects will also be observed after edoxaban pretreatment and PAR-2 knockdown. In addition, Kupffer cells also have an important role in acute hepatic inflammation; therefore, these cells should be evaluated. Further studies are required to clarify the true efficacy of edoxaban treatment for protecting against hepatic IRI.

## Conclusions

Edoxaban ameliorated hepatic IRI in mice by protecting against micro-thrombosis in sinusoids and suppressed FXa-PAR-2-induced inflammation in the SECs.

## Supporting information

S1 Fig Dose determination of the orally active prodrug of edoxaban (edoxaban tosylate monohydrate) *in vivo*. Edoxaban treatment demonstrated a cytoprotective effect in dose-dependent manner (n = 5 per group). Treatment with 50 mg/kg edoxaban significantly decreased serum ALT levels compared with the vehicle group. P values from one-way analysis of variance with the Welch test followed by the Games-Howell test. * *p* < 0.05.
(TIF)

S2 Fig Dose determination of the edoxaban in hypoxia/reoxygenation (H/R) model of sinusoidal endothelial cells (SECs). There was a significant difference among the groups (*p* = 0.008 by Kruskal-Wallis test). Although there was no statistical difference between the groups in multiple comparisons (Steel-Dwass test), 1μM of edoxaban seemed to be most effective in reducing LDH cytotoxicity in the H/R model of SECs (n = 4 per group).
(TIF)

S3 Fig The enhancement of PAR-2 expression in the liver after ischemia-reperfusion injury (IRI). (A) In real-time PCR analysis, PAR-2 expression was significantly increased after IRI *in vivo* (n = 5 per group). (B) Western blot analysis showed that PAR-2 generation was significantly increased after IRI *in vivo* (n = 5 per group). (C) In western blot analysis, PAR-2 generation was significantly increased after H/R *in vitro* (n = 5 per group). P-value from the Mann-Whitney U test.
(TIF)

S1 Raw images (PDF)

S1 Dataset (XLSX)
