# Supplementary figures and images for: Xa inhibitor edoxaban ameliorates hepatic ischemia-reperfusion injury via PAR-2–ERK 1/2 pathway
Source: PLoS One. 2024 May 15;19(5):e0292628. doi: 10.1371/journal.pone.0292628 (PMC11095713; doi:10.1371/journal.pone.0292628)

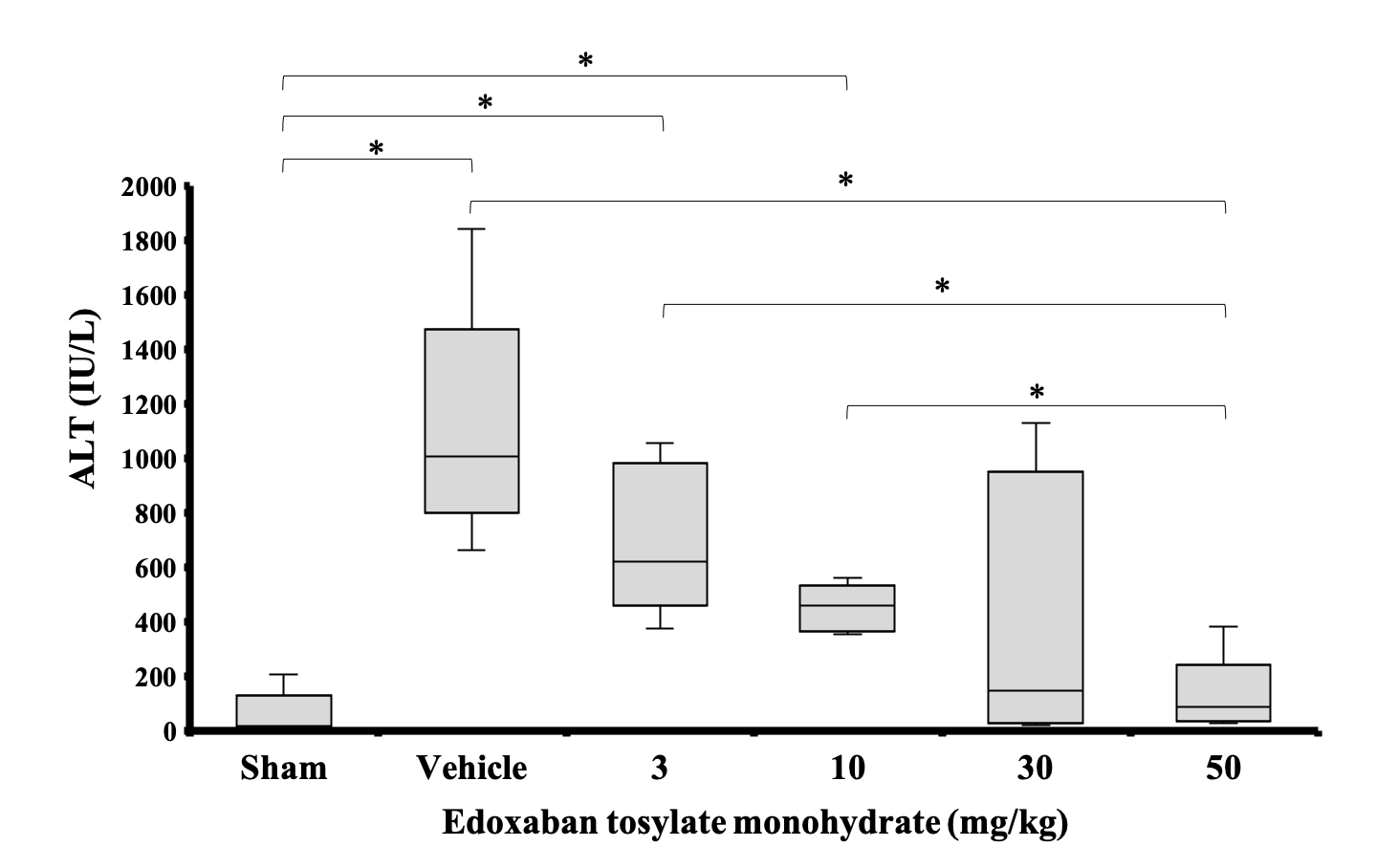

Supplement: S1 Fig — Edoxaban treatment demonstrated a cytoprotective effect in dose-dependent manner (n = 5 per group). Treatment with 50 mg/kg edoxaban significantly decreased serum ALT levels compared with the vehicle group. P values from one-way analysis of variance with the Welch test followed by the Games-Howell test. * p < 0.05. (TIF) [file pone.0292628.s001.tif]

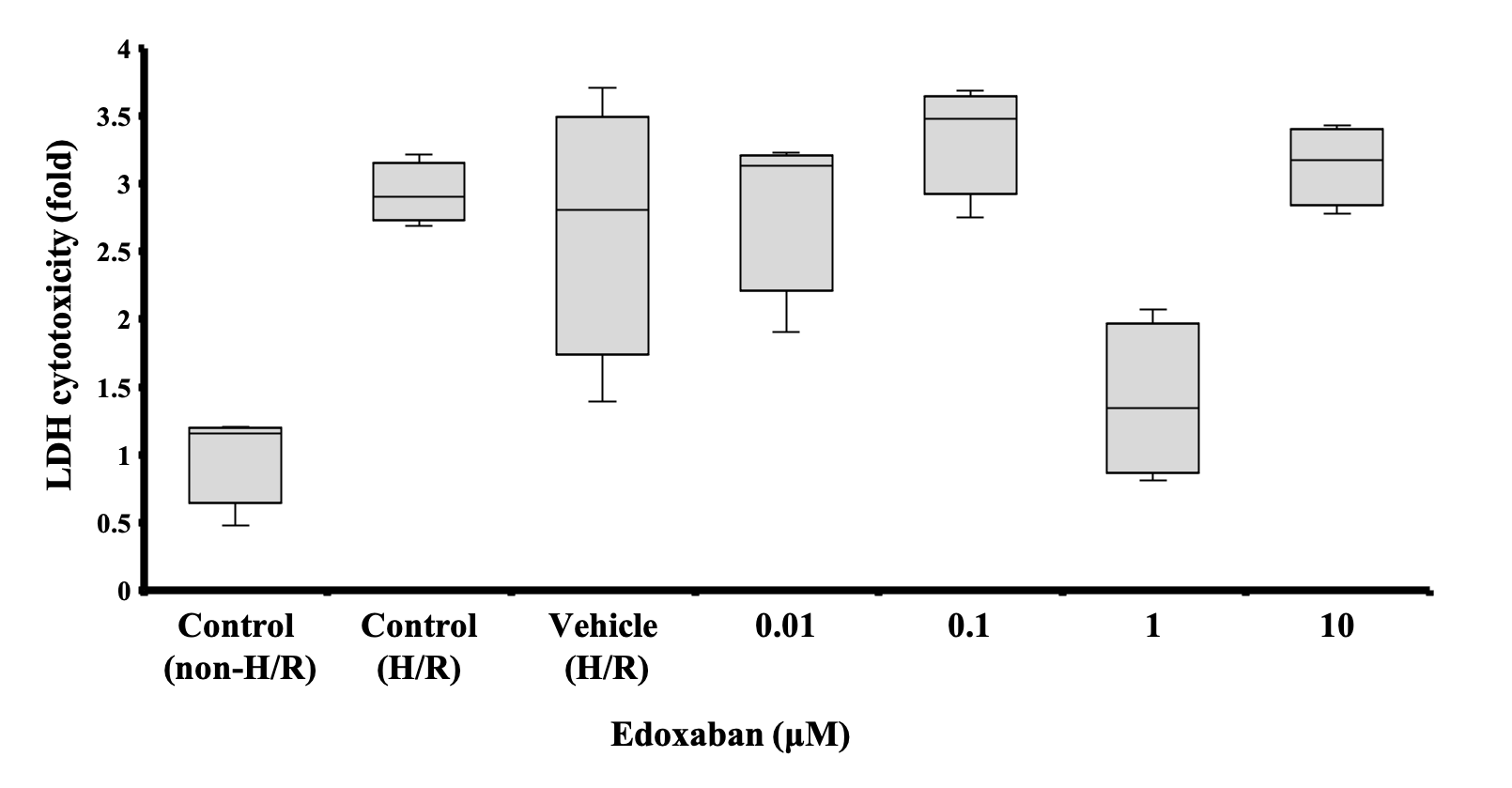

Supplement: S2 Fig — There was a significant difference among the groups (p = 0.008 by Kruskal-Wallis test). Although there was no statistical difference between the groups in multiple comparisons (Steel-Dwass test), 1μM of edoxaban seemed to be most effective in reducing LDH cytotoxicity in the H/R model of SECs (n = 4 per group). (TIF) [file pone.0292628.s002.tif]

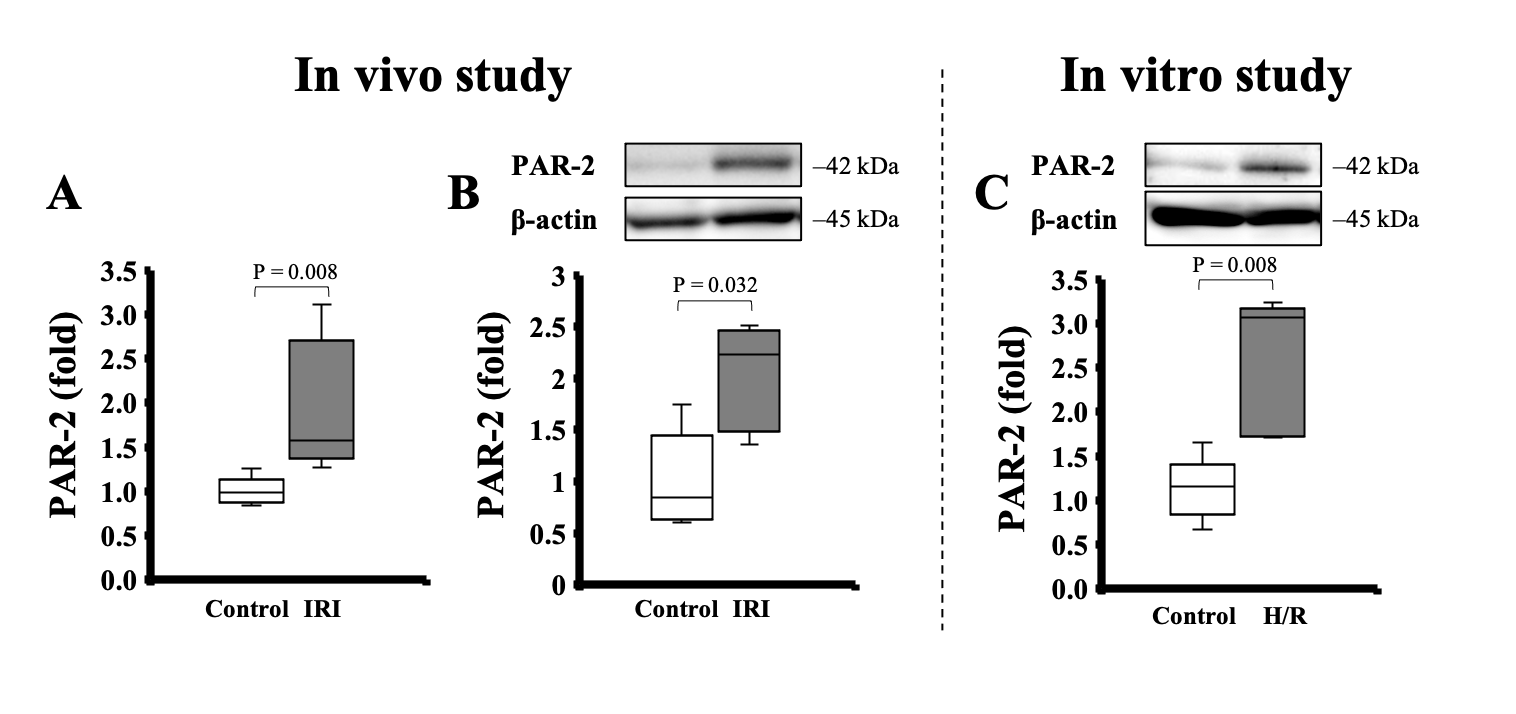

Supplement: S3 Fig — (A) In real-time PCR analysis, PAR-2 expression was significantly increased after IRI in vivo (n = 5 per group). (B) Western blot analysis showed that PAR-2 generation was significantly increased after IRI in vivo (n = 5 per group). (C) In western blot analysis, PAR-2 generation was significantly increased after H/R in vitro (n = 5 per group). P-value from the Mann-Whitney U test. (TIF) [file pone.0292628.s003.tif]
