# Supplementary material for: Xa inhibitor edoxaban ameliorates hepatic ischemia-reperfusion injury via PAR-2–ERK 1/2 pathway
Source: PLoS One. 2024 May 15;19(5):e0292628. doi: 10.1371/journal.pone.0292628 (PMC11095713; doi:10.1371/journal.pone.0292628)
Supplement: S1 Raw images — (PDF) [file pone.0292628.s004.pdf]

Fig.2A Fibrin

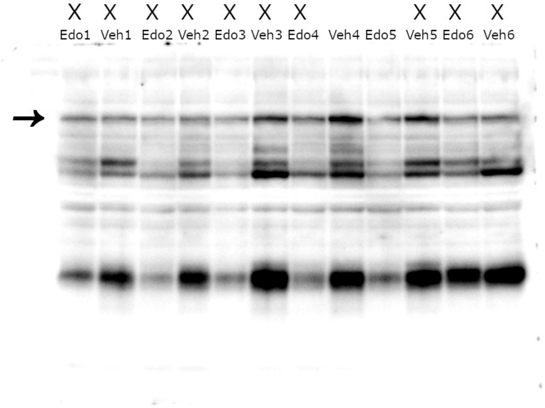

Fig.2A B-actin

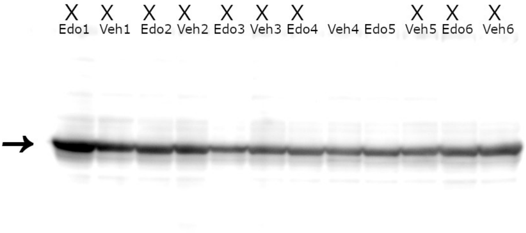

Fig.4D B_actin

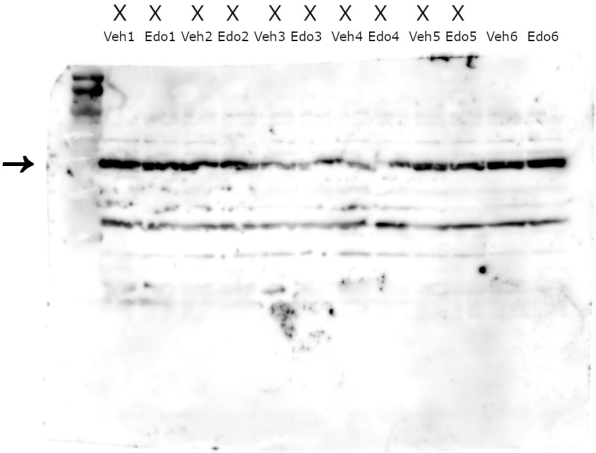

Fig.4D cleaved_caspase3

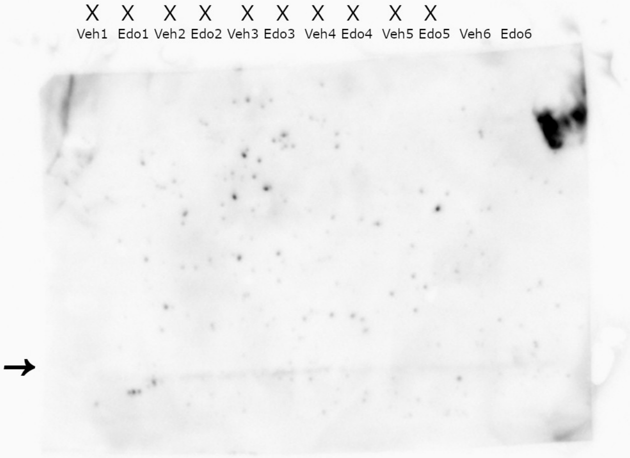

Fig.4D pro_caspase3

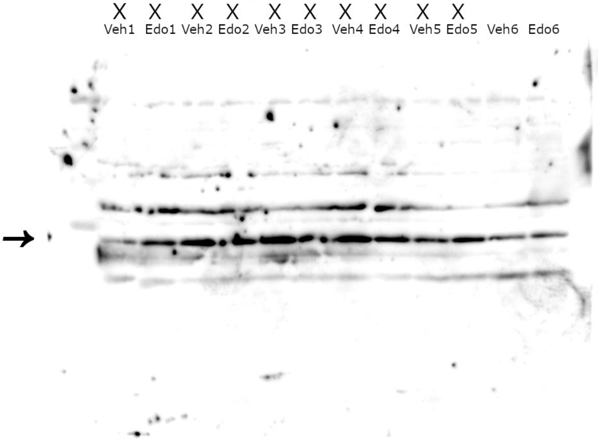

Fig.4E B-actin

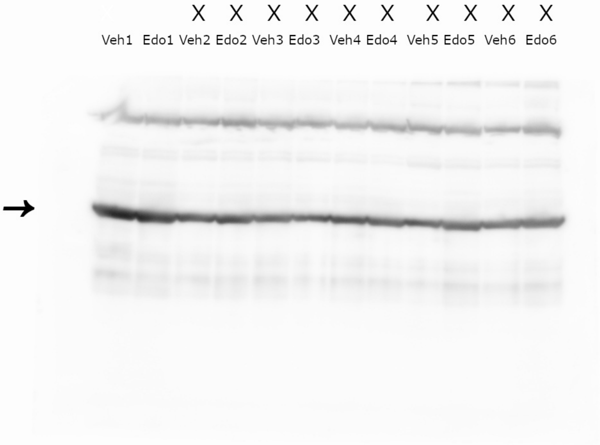

Fig.4E cleaved_caspase3

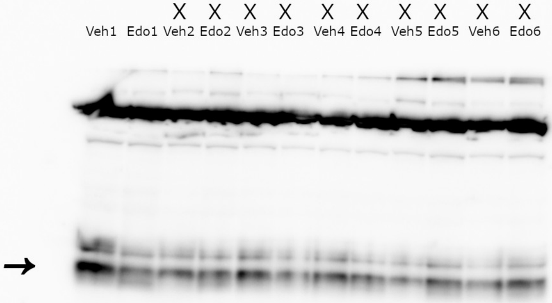

Fig.4E pro_caspase3

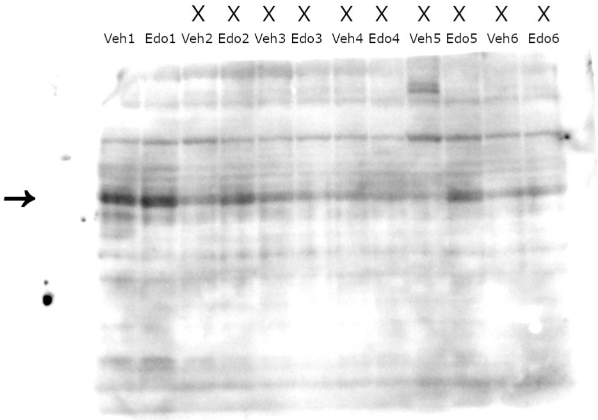

Fig.5A B-actin

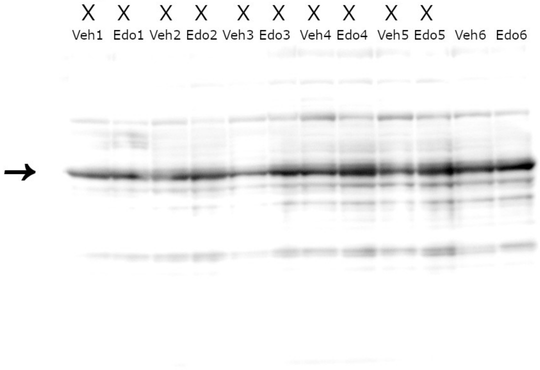

Fig.5A PAR-2

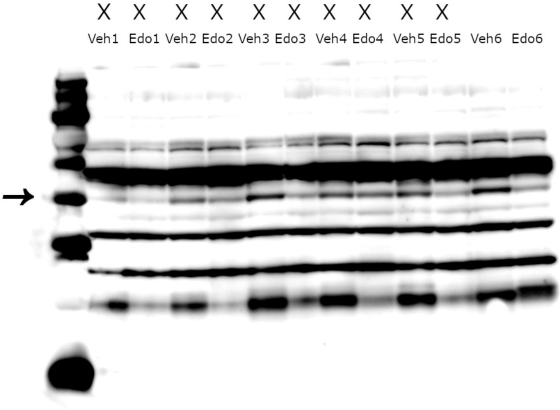
X X X X X X X X X X
Veh1 Edo1 Veh2 Edo2 Veh3 Edo3 Veh4 Edo4 Veh5 Edo5 Veh6 Edo6

Fig.5B phospho-ERK1/2

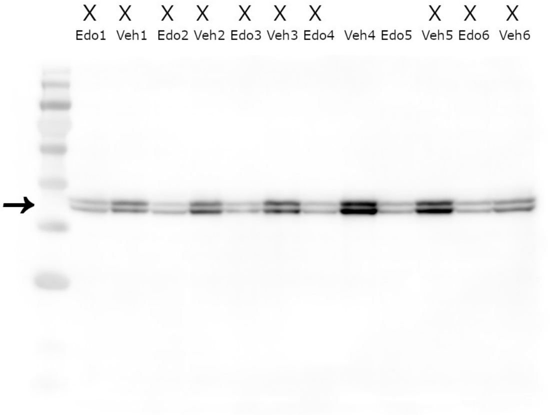

Fig.5B total-ERK1/2

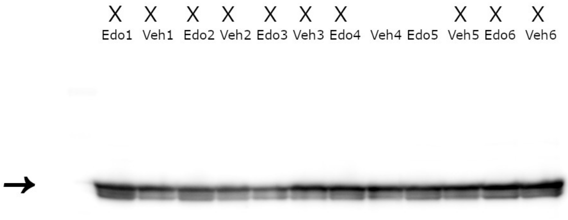

Fig.6D B-actin

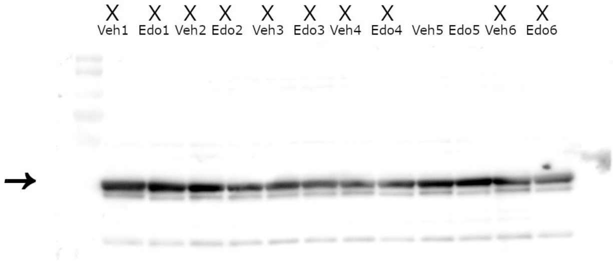

Fig.6D PAR-2

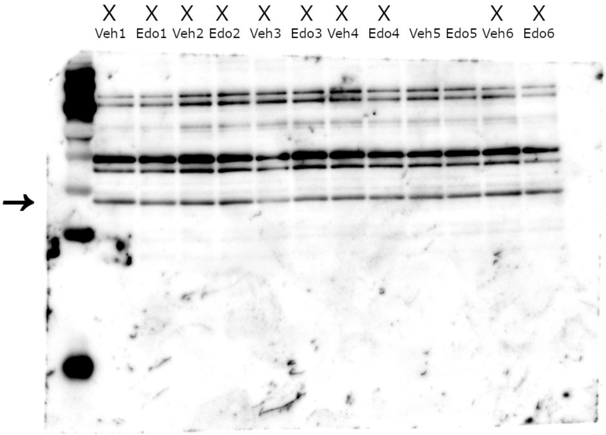
X X X X X X X X X X
Veh1 Edo1 Veh2 Edo2 Veh3 Edo3 Veh4 Edo4 Veh5 Edo5 Veh6 Edo6

Fig.6E B-actin

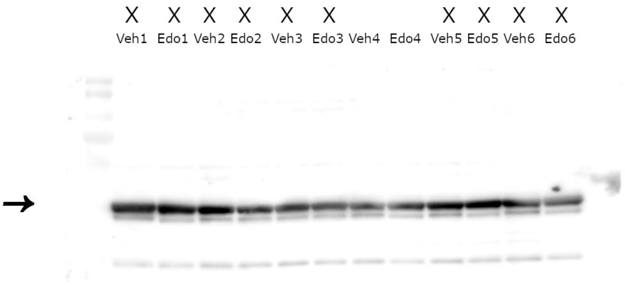

Fig.6E phosphoERK1/2

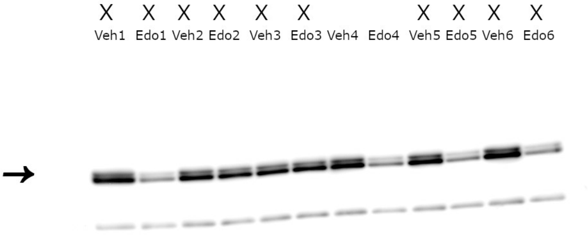

Fig.6E total ERK1/2

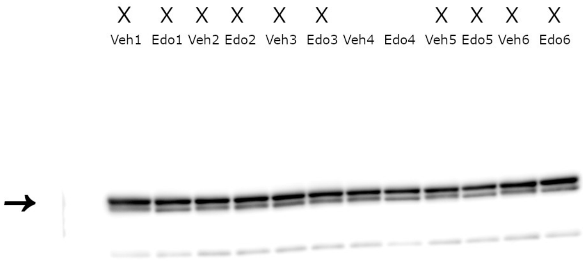

Fig.7A B-actin

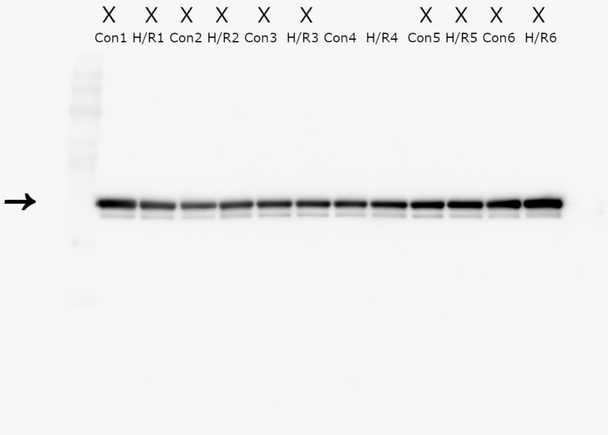

Fig.7A phospho ERK1/2

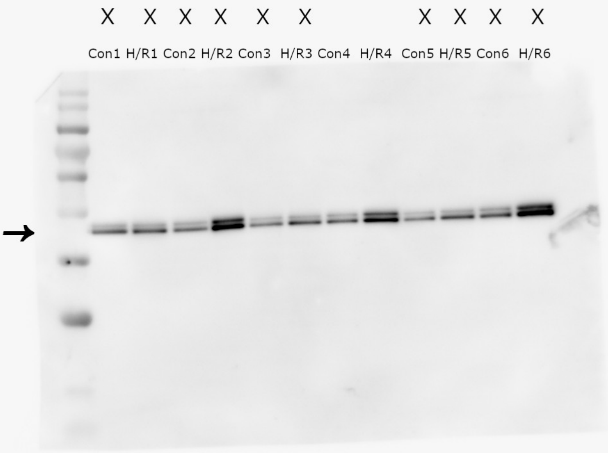

Fig.7A total ERK1/2

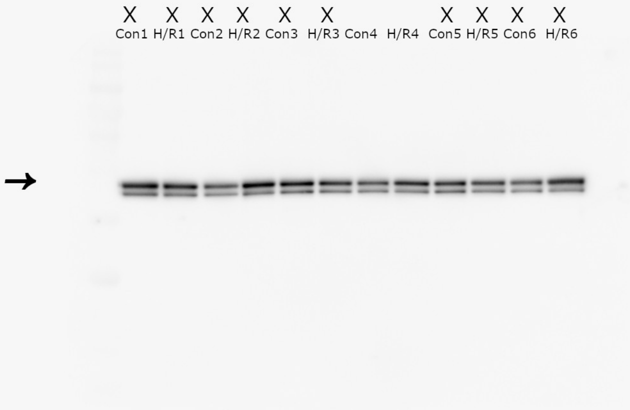

Fig.7CB-actin

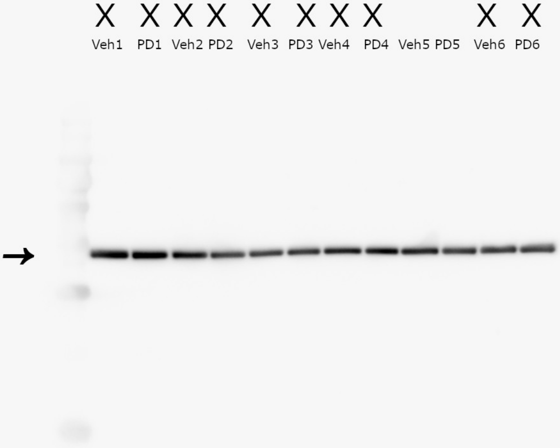

Fig.7C PAR-2

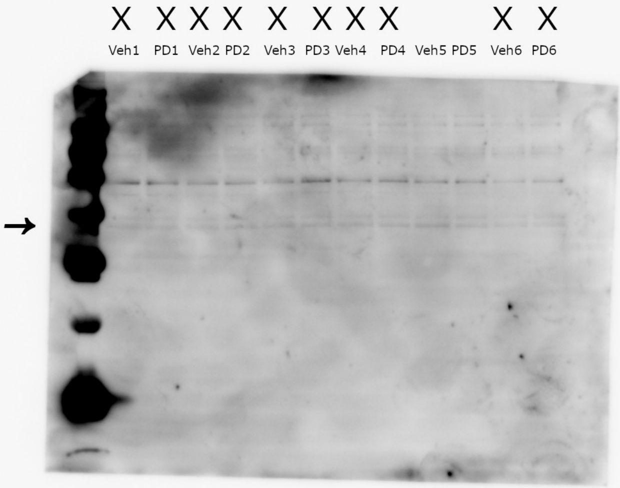
X X X X X X X X X X
Veh1 PD1 Veh2 PD2 Veh3 PD3 Veh4 PD4 Veh5 PD5 Veh6 PD6
→

Fig.7E B-actin

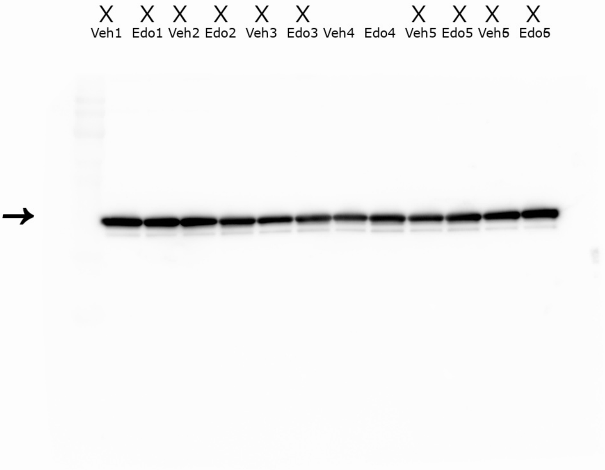

Fig.7E phospho ERK1/2

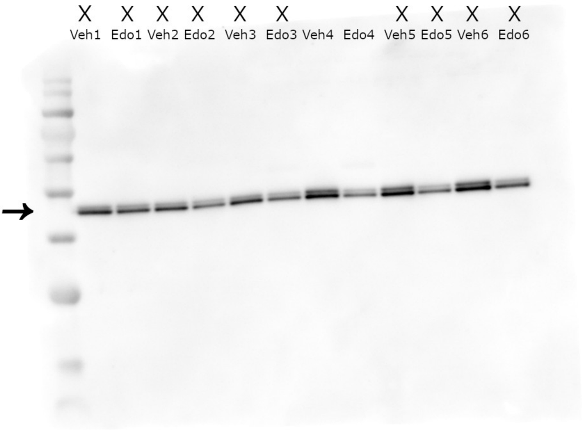

Fig.7E total ERK1/2

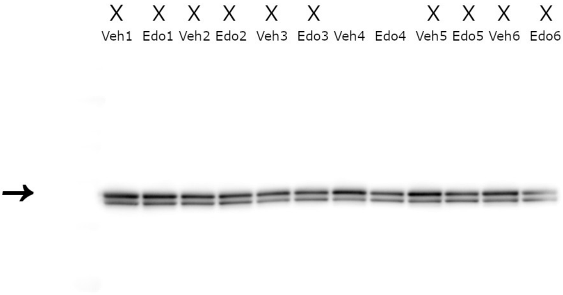

S3 fig.3B B-actin

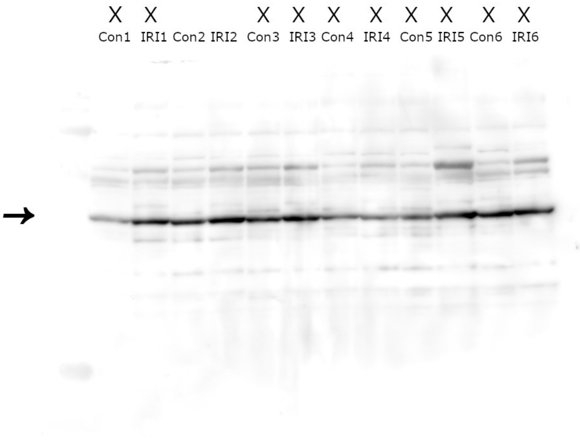

S3 fig.3B PAR-2

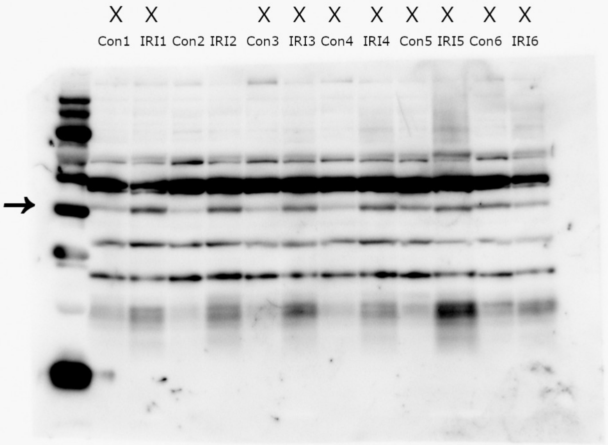

S3 Fig.C B-actin

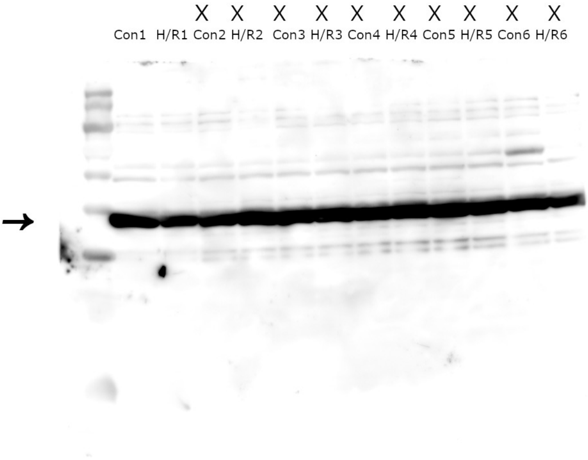

S3 Fig.C PAR-2

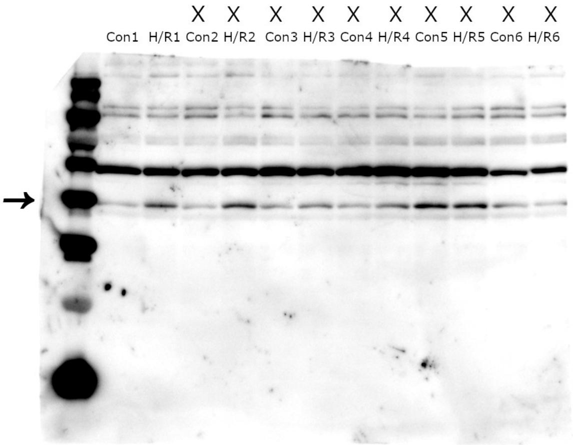
X X X X X X X X X X
Con1 H/R1 Con2 H/R2 Con3 H/R3 Con4 H/R4 Con5 H/R5 Con6 H/R6
